# Partial bicorporeal uterus with unexpected cervical findings and a longitudinal obstructing vaginal septum – a case report

**DOI:** 10.52054/FVVO.15.2.071

**Published:** 2023-06-30

**Authors:** H Ahmad, B Pouseele

**Affiliations:** Department of gynaecology, O.L.V. Van Lourdes Ziekenhuis, 8790 Waregem, Belgium

**Keywords:** Mullerian anomaly, case study, triple cervix, cervical anomaly, OHVIRA

## Abstract

Mullerian duct anomalies are prevalent in 4-7% of the female population and come in many different shapes and forms. A lot of effort has already gone into trying to classify these anomalies, and some are still found that do not match any of the subcategories. We report a 49-year-old patient, presenting with abdominal pressure and recent onset of abnormal vaginal bleeding. A laparoscopic hysterectomy was performed, which revealed a U3a-C(?)-V2 mullerian anomaly with three cervical ostia. The origin of the third ostium remains unclear. Early and correct diagnosis of Mullerian anomalies is of the utmost importance to provide individually tailored care and to avoid unnecessary surgeries.

## Introduction

Mullerian duct anomalies are defined as congenital malformations of the female genital tract stemming from embryological maldevelopment of the Mullerian ducts. They have a prevalence of 4-7% and are as such a rather common benign affliction. They consist of a myriad of conditions, depending on the level and severity of maldevelopment. Symptoms can vary from asymptomatic to extreme dysmenorrhea and fertility problems. Diagnosis of these malformations is mostly made by clinical examination and ultrasound, sometimes aided by hysterosalpingogram, hysteroscopy, MRI or laparoscopy. In most cases there is no need for therapeutic interventions, except to preserve fertility or to alleviate symptoms in the case of obstructive malformations (Grimbizis et al., 2013; Acién and Acién, 2016).

We present a rare case of a 49-year-old patient, complaining of abdominal pressure and abnormal vaginal bleeding. Diagnostic work-up did not reveal a clear cause. Following a hysterectomy, the pathology report revealed a Mullerian anomaly, which we classified as U3a-C(?)-V2 with three cervical ostia. Two of these ostia lead to two separate endometrial cavities; the third merely consisted of a rudimentary cervical canal opening into a rudimentary obstructed vaginal canal, which is extremely rare.

## Case report

The patient was a 49-year-old female complaining of abdominal pressure and recent onset of abnormal uterine bleeding.

Surgical history included a cholecystectomy, 3 Caesarean sections and a tubal ligation. Previous pelvic ultrasound had raised the possibility of a bicornuate uterus. A laparoscopy performed in 1985 had also suggested endometriosis. The patient also had a history of pulmonary embolism, and she was being treated with rivaroxaban due to a recent recurrence. Unfortunately, no operative reports were available from previous surgeries, nor any pre-operative ultrasound images.

The patient presented to the clinic complaining of abdominal pressure and abnormal uterine bleeding for 6 months, having previously being amenorrhoeic on the progestogen-only pill.

Clinical examination revealed a normal cervix and vaginal canal. Pelvic ultrasound revealed a suspected bicornuate uterus with a thin endometrium in both horns, no evidence of myomas and uterine measurements of 76mm x 34mm x 70mm. Only one ovary could be visualised. On the right side there was a multilocular cystic mass of unclear origin, measuring 77mm x 50mm x 51mm. There was no evidence of ascites on the pelvic ultrasound.

Abdominal CT scan revealed no signs of diverticulitis or other gastrointestinal pathology. No pathologically enlarged lymph nodes were observed.

An MRI was performed which revealed a Mullerian duct anomaly described as a bicornuate uterus and absent right kidney. The mass was described as a cystic or tubular structure on the right of the uterus, containing blood products. Suggested differential diagnoses included a dilated right fallopian tube containing blood, or a rudimentary right ureter. The right ovary could not be visualised.

After counselling, the patient consented to a laparoscopic total hysterectomy, with investigation and removal of the unknown mass.

During surgery, a bicornuate uterus, possibly bicorporeal was noted. The right ovary was present and normal in appearance, albeit very small. The left ovary was absent, presumably resected during earlier endometriosis surgery. The right ureter could not be visualised. Upon first inspection of the abdomen, no evidence of the unknown mass was found.

While performing a standard colpotomy, there was a sudden evacuation of a moderate amount of brown mucous fluid. Upon further inspection, we found a rudimentary closed off vaginal canal, which was also resected.

Inspection of the uterus directly after transvaginal extraction revealed a U3a-C(?)-V2 Mullerian anomaly with 3 cervical ostia.

Careful probing of the ostia with a hysterometer revealed that the left and right ostia led to an endometrial cavity. The middle cervical canal measured only 3 cm.

The pathology report confirmed the presence of two separate uterine cavities, each with their own cervical canal and cervix. The two hemi-uteri were fused. The report also confirmed the presence of an extra, intermediate cervical ostium, ending in a small, dilated cervical canal. Pathological analysis of the vaginal canal revealed signs of prolonged pressure and chronic inflammation. Findings were also consistent with uterine adenomyosis.

There were no immediate postoperative complications. At 4 and 8 weeks post-surgery, the patient reported relief of her preoperative symptoms. Permission to publish this case was obtained.

## Discussion

### Results

Preoperatively, we believed this to be a bicornuate uterus. However, after surgery our diagnosis had to be adjusted. Final diagnosis in this patient was a U3a-C(?)-V2 Mullerian anomaly with three cervical ostia and an obstructed hemivagina next to a normal vaginal canal. This is a rare form of OHVIRA syndrome (Obstructed Hemi-Vagina and Ipsilateral Renal Agenesis). Typically, OHVIRA is associated with a complete bicorporal uterus, though in this case there was only a partial divide.

The absence of symptoms or reproductive problems resulted in a late diagnosis at age 49, normally these kinds of anomalies are usually detected at an earlier stage of life. It is worth noting that the patient received a diagnosis of endometriosis at a very young age (15 years old), which could have been an indicator of retrograde menstrual bleeding, caused by occlusion of one hemi-uterus. No haematometra was ever observed in this patient, but the partial bicorporeal uterus could have resulted in a connection at the isthmic level, allowing drainage of both hemi-uteri. Renal agenesis was undiagnosed until presentation and imaging, and the extent of the patient’s Mullerian anomaly was only fully diagnosed during surgery. The reason for the onset of symptoms at a more advanced age is unclear.

**Figure 1:**
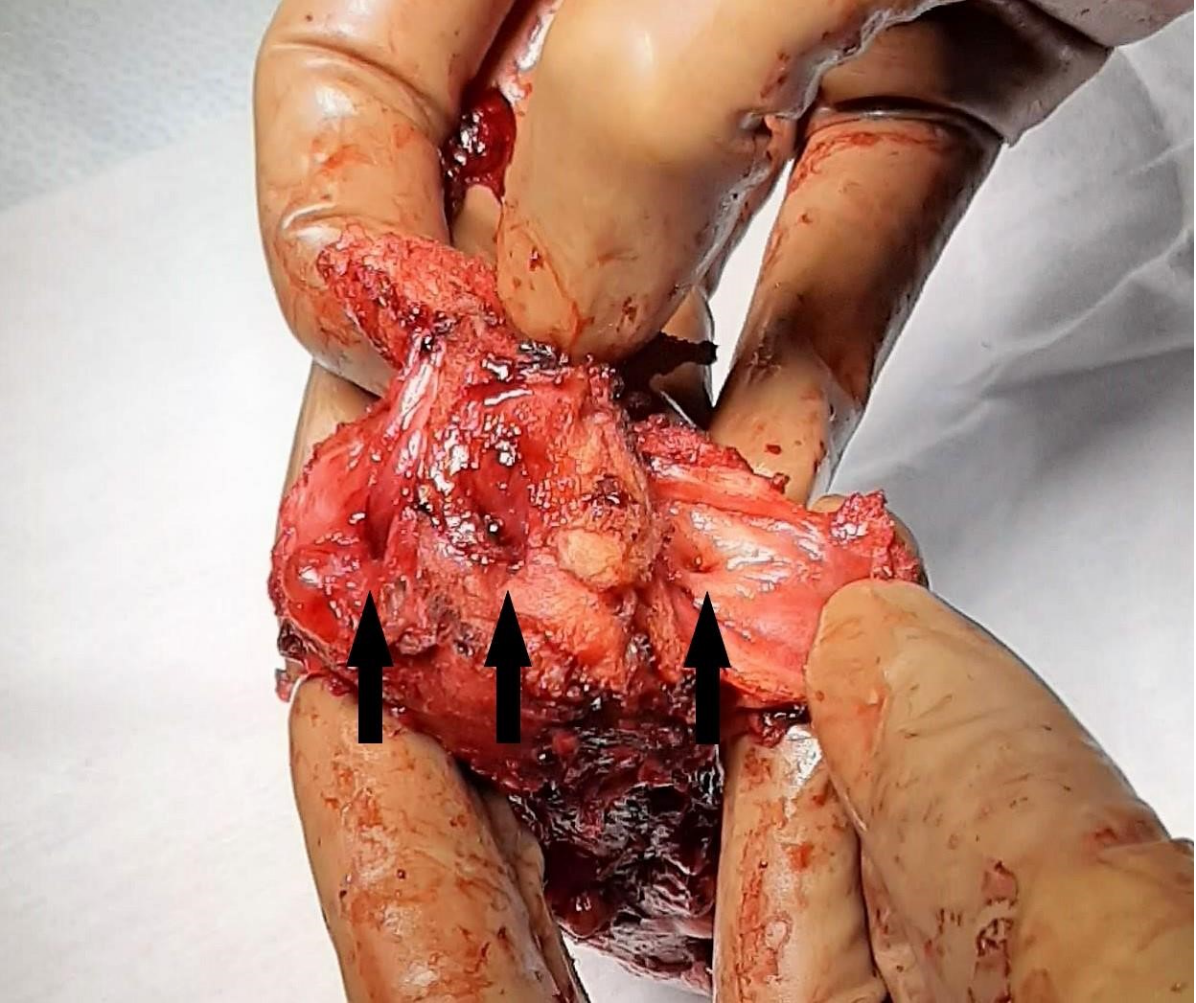
Identification of 3 cervical ostia.

**Figure 2:**
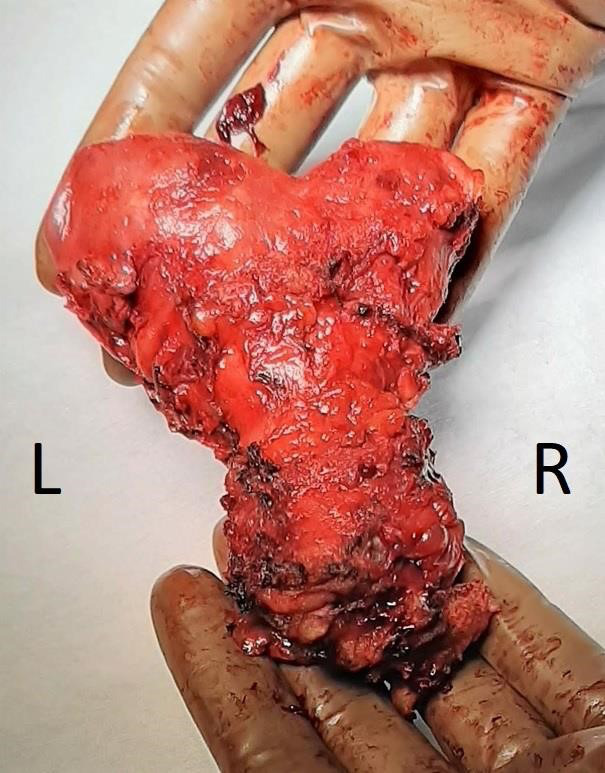
Uterine body.

**Figure 3:**
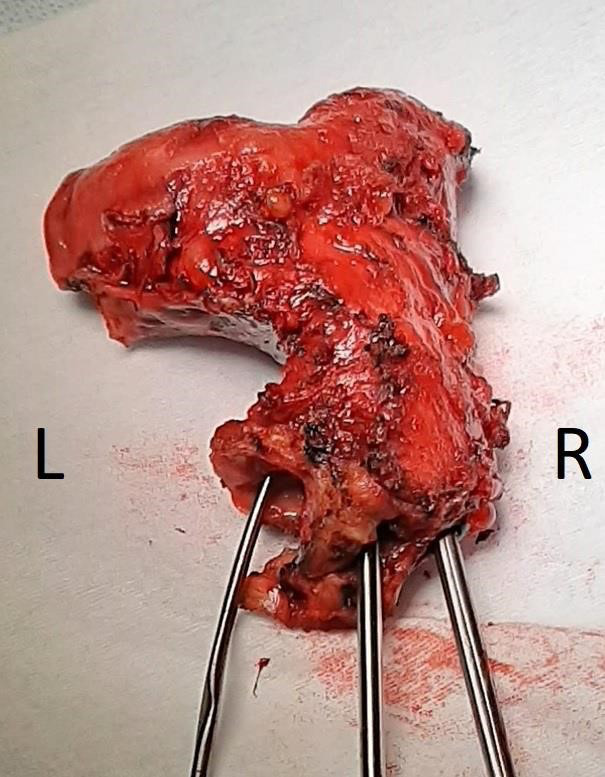
Uterine body with instruments inserted in three separate ostia.

**Figure 4:**
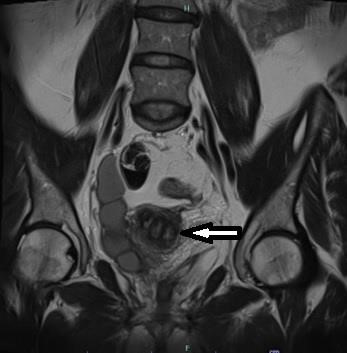
MRI showing three separate endocervical canals.

### Strengths and limitations

To the best of our knowledge, this is the first time that a case with possible triple cervices has been described. There are, however, several limitations to this case report. First and foremost, the specimen was not examined by a pathologist experienced in Mullerian anomalies.

Secondly, the absence of previous surgical reports is unfortunate. Lastly, our full diagnosis was obtained during surgery, so no hysteroscopy or hysterosalpingography could be performed. The extent of the Mullerian anomaly was possibly underestimated in this case, due to the fact that only one cervix was seen during the preoperative examination.

### Literature

This patient was diagnosed with right renal agenesis and a right obstructed hemi-vagina, which is also known as OHVIRA syndrome. This syndrome represents 7.1% of all female genital tract malformations. Differential diagnosis of the mass includes a Gartner’s cyst or ectopic ureter. However, given the uterine anomaly, renal agenesis, and the absence of urinary incontinence, OHVIRA syndrome is most likely. A higher prevalence of endometriosis in these patients has been described, which was indeed also present in this patient’s history. (Acién and Acién, 2016). Studies in rat embryos suggested a Wolffian origin of the vagina, which would explain the frequent association of renal agenesis with vaginal abnormalities. This study also showed that distal to the fusion of the Müllerian ducts, they diverge again before fusing with the Wolffian ducts to end in the sinus bulbs. The authors postulate that this distal diverging would correspond to the internal and external cervical ostium in rats (Sánchez- Ferrer et al., 2006).

A differential diagnosis is more difficult to establish for the third cervical ostium. A fistula following previous Caesarean section seems less likely, considering that the cervical canal did not connect to either of the endometrial cavities or the abdominal cavity. Furthermore, the third ostium and endocervical canal matched the histological properties of the two other normal cervices. A Gartner’s pseudocyst could also be considered, but this would mean that one cervix was atretic, with no connection to the endometrial cavity. However, this patient had two different, distinct cervices with a clear connection to their respective endometrial cavities. Another very rare possibility is duplication of Mullerian tract tissue at the level of the cervix (Acién and Acién, 2016). An atretic ectopic ureter could be considered, especially given the theory stated above, that the Mullerian and Wolffian tracts fuse just below the level of the cervix. However, then the third blind ostium should have been placed laterally, instead of in between the other two ostia as seen in this patient (Acién et al., 2004).

No literature references were found for a triple cervical canal therefore it is not possible to estimate the prevalence of this Mullerian anomaly.

It is not even possible to provide a full classification of this specimen using the ESHRE (European Society of Human Reproduction and Embryology). The uterus can best be categorised as class U3, as it is a uterus with an abnormal fundal outline. Yet, our pathology report described the specimen as a uterus didelphys: two separate cavities with fusion of the hemi-uteri, making this a class U3b. Macroscopically however, the divide seemed to be above the level of the cervices, so we would classify this as U3a. The vaginal septum was longitudinal and obstructing, which would be sub-class V2. The cervices, however, don’t fit any of the subclasses. Using the ESHRE classification, this would be U3aC?V2 (Grimbizis et al., 2013).

The ASRM (American Society for Reproductive Medicine) developed a new classification system in 2021, based on the 1988 AFS (American Fertility Society) system. In this new system, they stress that not all possible anomalies are included. Given the variety and complexity of Mullerian anomalies, it is likely that more will have to be added in the future. They describe possible variations on the uterine malformations with obstructed hemi-vagina, none of which suggest the existence of triple cervices. They do, however, make note of the association between ipsilateral renal agenesis and obstructing longitudinal vaginal septum. It is possible that only one cervix will be observed during clinical examination due to this septum. Imaging options include ultrasound, MRI and HSG, but in our case the third cervical canal was not observed on ultrasound or MRI. However, further inspection of the MRI images after the surgical findings, reveals that a triple canal cervix can be visualised (Pfeifer et al., 2021).

### Clinical impact and future perspectives

The clinical impact of a possible triple cervix is unclear. In this case, the patient had no problem conceiving. She did, however, experience symptoms of endometriosis at a very early age, and delivered her children through Caesarean section, though the reason for this was unknown.

Early and correct diagnosis of these types of Mullerian anomalies is of the greatest importance to identify other cases, and to reduce the risk of severe endometriosis. Renal agenesis should always raise the suspicion of Mullerian anomalies and instigate further examination. MRI imaging, hysteroscopy and HSG could shed more light on the extent and severity of the suspected anomaly. Using this approach, perhaps symptoms can be alleviated using minimally invasive surgery (Acién and Acién, 2016).

Our patient specifically requested a hysterectomy and did not want to continue exploring non-surgical options. If this had been a patient desiring to get pregnant, a transvaginal resection of the vaginal septum could have been considered (Pfeifer et al., 2021).

## Conclusion

In conclusion, firstly, this is a unique case where there are three suspected cervical canals. This could raise questions about the embryological development of the cervix. Given the absence of fertility problems and lack of symptoms in earlier stages of life, this phenomenon may be under reported. The need for therapeutic intervention is unclear.

Secondly, in a patient with unilateral renal anomaly or agenesis, one must always consider the possibility of an associated Mullerian anomaly. Correct preoperative diagnosis could contribute to avoiding the need for major surgery.
